# Aptamer Sensors

**DOI:** 10.3390/bios7010005

**Published:** 2017-01-04

**Authors:** Giovanna Marrazza

**Affiliations:** Department of Chemistry “Ugo Schiff”, University of Florence, 50019 Sesto Fiorentino, Italy; giovanna.marrazza@unifi.it; Tel.: +39-055-4573-320

**Keywords:** aptamer, sensor, aptasensor, affinity sensor, biomimetic recognition element, electrochemical, optical, electrochemiluminescent, surface plasmon resonance

## Abstract

In the last years, great progress has been accomplished in the development of aptamer sensors with different transducers. In order to improve the sensitivity of these biosensors, several methodologies have been employed. In this Special Issue, the state of art and the future trends in the field of aptamer sensors have been explored.

## 1. Introduction

Nucleic acid-based aptamers are single-stranded DNA or short RNA sequences, isolated in vitro from a library of synthetic oligonucleotides using an automated technique named SELEX (selection evolution of ligands by exponential enrichment), first described in 1990. Oligonucleotides from the library are selected according to their affinity toward a target molecule, looking for the best selectivity and specificity. Aptamers, since their discovery, have attracted the attention of researchers. They started as therapeutic agents and in a few years they become a hot subject in analytical chemistry as well.

They have been quickly used in the sensors field as novel recognition elements due to their advantages with respect to antibodies such as high stability, low dimension and high affinity for small molecules. In addition, they are easy to synthesize in vitro, simple to modify and flexible to design in the sequences. These inherent benefits of oligonucleotides can not only contribute to target diversification but can also improve detection by efficient, exceptional binding constants (in the micromolar to picomolar range). At the same time, they can help analysts to assemble smart and convenient sensing platforms. For all these reasons, a large number of aptamer sensors have been rationally designed and various techniques have been used or combined to obtain a satisfactory detectable signal (optical, electrochemical, piezoelectric, etc.).

This current Special Issue shares promising strategies of successful aptamer sensors and consequently aims to motivate researchers to identify and investigate future applications in environmental, food and clinical analysis.

Menger et al. [1] focused their attention on the preparation and performances of aptamers used for the recognition of proteins and as enzyme mimics. Furthermore, the authors compared the chemical characteristics of aptamers to other synthetic receptors such as the molecular imprinted polymers (MIPs). The highly reproducible production of aptamers by chemical synthesis is a clear advantage while the preparation of MIPs typically leads to polymers with a distribution of different binding sites and differences between the batches of preparations. Improvement is expected from an enhanced functional monomer library combined either with a rational design or with an empirical approach associated with high-throughput synthesis and detection platforms. As compared with antibodies and aptamers, MIPs have been established only for a restricted spectrum of proteins. The authors concluded that for aptamers and MIPs, the measurement of groups of chemically similar substances or, alternatively, the indication of different analytes by only one recognition species would give additional advantages for comparison with the biological features.

P. K. R. Kumar [2] explored the evolution of aptamer sensors towards direct detection of intact viruses. Viral diagnosis plays an important role in understanding epidemics and in containment of disease by appropriate therapeutic interventions using specific antiviral drugs. Usually, it is performed by indirect serological methods, including the complement fixation test, the hemagglutination inhibition test, immunofluorescence, the enzyme linked immunosorbent assay and the Western blot assay. Although these assays are useful for viral diagnosis, they are laborious and time-consuming, possibly leading to delays in identifying the infectious agent and the treatment. Moreover, the serological diagnostic methods are less suitable for identifying newly emerging viral diseases. To overcome these limitations, biosensor-based platforms with various design strategies have been realized. In this review, aptamer-, antibody- and glycan-based coupled with different sensing platforms have been explored.

In recent years, whispering gallery mode resonators (WGMRs) have attracted increasing attention and a growing number of papers have been published, investigating structures from the simplest ones, namely cylindrical optical fibers and microspheres, to more complex fiber coils and bubble microresonators. Therefore, WGMRs have different geometries with unique spectral properties, including narrow linewidth, high stability, and good tunability. Up to now, the WGMRs that have been used as aptamer sensors are silica microspheres and silicon oxynitride (SiON) ring resonators. The surface functionalization, the experimental set-ups and the applications of this kind of sensor have been described by Nunzi Conti et al. [3]. In particular, aptamer sensors for the detection of thrombin and vascular endothelial growth factor biomarkers and aflatoxin M1 have been described.

Ravalli et al. [4] reviewed the state of the art and the new trends of aptamer sensors for protein biomarker analysis using electrochemichemistry electrochemiluminescence and photoelectrochemical transduction methods.

In the last decades, nanomaterials have found wide applications in sensor technology because they can greatly facilitate the miniaturization of instrumentation and improve the analytical performance; in addition, they have unique electrochemical properties. In fact, nanostructures increase the electron transfer between the redox center and the electrode surface. Moreover, the number of immobilized bioreceptors increases because of their high surface/volume ratio. A special focus has been placed on the nanostructure sensors by the authors.

A new generation of aptamer-based methods for gluten quantification is explored by Miranda-Castro et al. [5]. Although still in its infancy, this sensitive technology will undoubtedly continue to advance. It is reasonable to expect that detailed, comprehensive studies to obtain extensive molecular-level information about the aptamer-protein and aptamer-peptide interactions could allow for a better understanding of the structural basis of this highly specific interaction, leading to new, modified aptamers with improved affinity properties.

## 2. Conclusions

The choice of receptors for use as biosensor components is of vital importance and will affect the performance of the sensor. For this reason, researchers in this field should determine all essential features that need to be assessed in each specific application.

The achievement of the papers submitted to this Special Issue confirms the importance of aptamer sensors in a wide variety of the biosensor technology. The reported knowledge will encourage active researchers and will contribute to further progress in the field.
